# Bats as Hosts of Antimicrobial-Resistant *Mammaliicoccus lentus* and *Staphylococcus epidermidis* with Zoonotic Relevance

**DOI:** 10.3390/vetsci12040322

**Published:** 2025-04-01

**Authors:** Vanessa Silva, Manuela Caniça, Rani de la Rivière, Paulo Barros, João Alexandre Cabral, Patrícia Poeta, Gilberto Igrejas

**Affiliations:** 1 LAQV-REQUIMTE, Department of Chemistry, NOVA School of Science and Technology, Universidade Nova de Lisboa, 1099-085 Caparica, Portugal; 2 Department of Genetics and Biotechnology, University of Trás-os-Montes and Alto Douro (UTAD), 5000-801 Vila Real, Portugal; 3 Functional Genomics and Proteomics Unit, University of Trás-os-Montes and Alto Douro (UTAD), 5000-801 Vila Real, Portugal; 4 Microbiology and Antibiotic Resistance Team (MicroART), Department of Veterinary Sciences, University of Trás-os-Montes and Alto Douro (UTAD), 5000-801 Vila Real, Portugal; 5 National Reference Laboratory of Antibiotic Resistances and Healthcare Associated Infections, Department of Infectious Diseases, National Institute of Health Dr. Ricardo Jorge, 1649-016 Lisbon, Portugal; 6 Centre for the Studies of Animal Science, Institute of Agrarian and Agri-Food Sciences and Technologies, University of Porto, 4099-002 Porto, Portugal; 7 Associate Laboratory for Animal and Veterinary Sciences (AL4AnimalS), 5000-801 Vila Real, Portugal; 8 Centre for the Research and Technology of Agro-Environmental and Biological Sciences (CITAB), University of Trás-os-Montes and Alto Douro (UTAD), 5000-801 Vila Real, Portugal; 9 CECAV—Veterinary and Animal Research Centre, University of Trás-os-Montes and Alto Douro (UTAD), 5000-801 Vila Real, Portugal

**Keywords:** bats, *Mammaliicoccus lentus*, *Staphylococcus epidermidis*, antimicrobial resistance, zoonotic potential, one health

## Abstract

Bats are increasingly recognized as reservoirs for antimicrobial-resistant bacteria, playing a potential role in the dissemination of resistance genes across species and regions. In this study, 105 bats from 19 species in Portugal were sampled to investigate the presence, antimicrobial resistance, and genetic characteristics of *Mammaliicoccus* and *Staphylococcus* isolates. Thirteen *Mammaliicoccus lentus* and *Staphylococcus epidermidis* were recovered. Antimicrobial susceptibility testing revealed multidrug resistance in three isolates, with *S. epidermidis* carrying *mph*(C), *msr*(A), and *dfr*C genes, and *M. lentus* harboring *sal*B, *tet*(K), and *str*. Notably, *qac*A was detected in *S. epidermidis*, highlighting its plasmid-associated potential for horizontal gene transfer to more pathogenic bacteria. Heavy metal resistance genes (*ars*B and *cad*D) were also identified, suggesting the role of environmental factors in co-selecting antimicrobial resistance. Molecular typing revealed the *S. epidermidis* strain as ST297, a clone associated with both healthy humans and invasive infections. These findings emphasize the need for monitoring bats as reservoirs of resistance determinants, particularly in the context of zoonotic and environmental health. The presence of mobile genetic elements and plasmids further underscores the potential for the dissemination of resistance. This study reinforces the importance of adopting a One Health approach to mitigate the risks associated with antimicrobial resistance.

## Simple Summary

Bats are unique animals that play an important role in ecosystems, but they can also carry bacteria that are resistant to antibiotics. This study examined bats in Portugal to identify bacteria that might pose a risk to humans or other animals. We found two species of bacteria, *Mammaliicoccus lentus* and *Staphylococcus epidermidis*, some of which carried antibiotic-resistance genes. These bacteria could potentially spread their resistance to other, more harmful bacteria. Additionally, we found genes that allow the bacteria to resist certain environmental toxins, like heavy metals, which may confer cross-resistance to antibiotics. Understanding how bats carry and spread these bacteria helps scientists and public health officials assess risks and plan measures to prevent the spread of antibiotic-resistant bacteria. This research highlights the importance of monitoring wildlife to protect both human and animal health.

## 1. Introduction

Bats, belonging to the order Chiroptera, represent the second-largest mammalian order after rodents and exhibit remarkable physiological and ecological diversity. With over 1400 species distributed across nearly all regions of the world, they demonstrate impressive adaptability to various environments [1]. Many species spend daylight hours roosting in natural shelters, such as foliage, caves, tree hollows, and rock crevices, as well as in artificial structures. At night, they are highly active, feeding on a diverse range of food sources [2]. Bats are also increasingly recognized as reservoirs and carriers of numerous microorganisms, including viruses with significant pathogenic potential for humans. Despite harboring these pathogens, bats often display remarkable immunity to their effects [3,4]. While the public health implications of bat-associated viruses have received considerable attention, the role of bats in the ecology of bacterial pathogens remains less explored. Several studies have identified potentially zoonotic bacterial pathogens in bats worldwide, with some instances of bat-to-human transmission documented [5,6,7,8,9,10,11]. However, the true extent of such transmission events remains unclear, largely due to insufficient surveillance efforts.

Pathogen transmission from bats to humans, wildlife, or domestic species can occur through various routes. These include direct contact during ecotourism activities like cave exploration, bat hunting, and the consumption of contaminated food products such as fruits partially eaten by bats [3,12,13,14]. Additionally, the unique characteristics of bats, such as their role as asymptomatic pathogen reservoirs, their longevity, torpor, and migratory habits, make them efficient hosts for pathogen dispersal over vast geographic areas [15]. Urbanization and habitat loss further exacerbate the potential for pathogen spillover. As natural roosting sites diminish, bats increasingly interact with human populations, leading to heightened stress and weakened immune defenses. This can increase the risk of pathogen transmission among bats and between bats and humans [16,17]. However, the actual transmission of antimicrobial resistance genes from bats to humans remains a complex process that requires further investigation. While bats may act as reservoirs of resistant bacteria, the direct transfer of resistance genes would likely occur through environmental contamination, indirect contact with contaminated surfaces, or intermediary hosts such as livestock and vectors (e.g., insects) [18,19].

Given the interconnectedness of human, animal, and environmental health, the One Health approach offers a holistic framework for understanding and mitigating the risks associated with bat-associated pathogens. This integrative strategy emphasizes the need for cross-sectoral collaboration to prevent the emergence and spread of antimicrobial-resistant bacteria and to safeguard existing medical interventions [20]. One Health also prioritizes zoonotic diseases and AMR, as demonstrated by their inclusion among the key topics discussed during the FAO-OIE-WHO (Food and Agriculture Organization—World Organization for Animal Health—World Health Organization) tripartite meetings [21,22].

Among bacterial pathogens, staphylococci provide an interesting model for One Health studies due to their ability to adapt across different ecosystems. The *Staphylococcaceae* family includes nine genera, notably *Staphylococcus,* and the newly categorized *Mammaliicoccus* genus. The latter comprises five species previously grouped under the *Staphylococcus sciuri* cluster, namely *M. sciuri*, *M. lentus*, *M. vitulinus*, *M. stepanovicii*, and *M. fleurettii* [23]. Coagulase-negative staphylococci and mammaliicocci have garnered significant public health interest due to their association with a range of human infections, from mild skin conditions to severe diseases like sepsis and endocarditis [24].

Recent research has identified the presence of both staphylococci and mammaliicocci species in wild bats, with some strains exhibiting antimicrobial resistance. However, studies on bats from Europe remain scarce [24,25,26,27]. Further research is essential to understand the role of bats in the dynamics of zoonotic pathogens and antimicrobial resistance. Therefore, we aimed to isolate staphylococci and mammaliicocci from wild bats and to investigate their antimicrobial resistance profile, virulence, genetic lineages, and mobile genetic elements.

## 2. Materials and Methods

### 2.1. Sample Collection and Bacterial Isolates

A total of 105 bats were sampled for this study during bat capture procedures conducted for banding purposes between May to September 2022 in 9 locations in Portugal. Samples were collected using a swab applied to the mouth, the external surface of the nose, and the skin of the bats. The captured bats belonged to 19 different species: *Plecotus austriacus* (*n* = 20), *Pipistrellus pipistrellus* (*n* = 16), *Nyctalus leisleri* (*n* = 10), *Pipistrellus kuhlii* (*n* = 10), *Myotis escalerai* (*n* = 8), *Tadarida teniotis* (*n* = 8), *Myotis bechsteinii* (*n* = 6), *Miniopterus schreibersii* (*n* = 5), *Rhinolophus mehelyi* (*n* = 5), *Rhinolophus ferrumequinum* (*n* = 2), *Plecotus auratus* (*n* = 2), *Hypsugo savii* (*n* = 2), *Myotis mystacinus* (*n* = 2), *Myotis myotis* (*n* = 2), *Myotis daubentonii daubentoniid* (*n* = 2), *Myotis daubentonii nat* (*n* = 2), *Barbastella barbastellus*, *Myotis daubentoniid*, and *Rhinolophus euryale*. Detailed information on each sampled bat is available in Supplementary Table S1. All samples were identified and sent to the laboratory within 24 h of collection. The swabs were inserted in tubes with Brain Heart Infusion (BHI) broth supplemented with 6.5% NaCl and incubated at 37 °C for 18–24 h. Then, the inoculum was seeded onto Mannitol Salt agar and ChromAgar MRSA plates and incubated at 37 °C for 24 and 48 h, respectively. The species of the isolates was confirmed by matrix-assisted 270 laser desorption/ionization–time-of-flight mass spectrometry (MALDI-TOF MS, Bruker, Ettlingen, Germany).

### 2.2. Antimicrobial Susceptibility Testing

The antimicrobial susceptibility of the isolates was assessed using the disk diffusion method, following the guidelines established by the European Committee on Antimicrobial Susceptibility Testing (EUCAST, 2018). An exception was made for kanamycin, which was evaluated according to the *American Clinical & Laboratory Standards Institute* (CLSI, 2017) guidelines. The concentrations used per disk (Liofilchem, Italy) were as follows: penicillin (1U), cefoxitin (30 μg), tetracycline (30 μg), linezolid (10 μg), trimethoprim/sulfamethoxazole (1.25/23.75 μg), ciprofloxacin (5 μg), erythromycin (15 μg), clindamycin (5 μg), gentamicin (10 μg), tobramycin (10 μg), chloramphenicol (30 μg), fusidic acid (10 μg), kanamycin (30 μg), and mupirocin (μg). The *S. aureus* strain ATCC 25923 was included as a quality control reference in the susceptibility testing.

### 2.3. Whole Genome Sequencing

Whole Genome Sequencing (WGS) was conducted using a NextSeq 2000 Illumina platform. Quality control of the reads and de novo assembly were carried out with INNUca v4.2.2 (https://github.com/B-UMMI/INNUca) (accessed on 28 February 2024). Genome annotation was performed using Prokka v1.16.6 (https://doi.org/10.1093/bioinformatics/btu153) (accessed on 28 February 2024). The average nucleotide identity (ANI) was determined using fastANI v1.33 (https://github.com/ParBLiSS/FastANI) (accessed on 28 February 2024).

Subsequently, the WGS data were analyzed to assess the antibiotic-resistance profiles using abricate v1.0.1 (https://github.com/tseemann/abricate#citation) (accessed on 28 February 2024) with the CARD and NCBI AMRFinder databases. Multilocus Sequence Typing (MLST) was applied to classify the isolates into sequence types and clonal complexes. Additionally, virulence genes were identified using abritamr v1.0.14 (https://www.nature.com/articles/s41467-022-35713-4) (accessed on 28 February 2024) alongside the pre-downloaded VFDB database in abricate.

## 3. Results and Discussion

Wildlife serves as a significant reservoir for numerous bacterial pathogens, many of which exhibit high levels of antimicrobial resistance. These animals play a crucial role in the transmission and dissemination of zoonotic bacteria to humans and other species. Among them, bats are of particular interest due to their adaptability to human-modified environments and their frequent exposure to diverse ecological niches, including contaminated habitats [28]. Bats’ ability to travel long distances further enhances their role as potential vectors in the spread of resistant bacteria across regions [29]. Their close interactions with other wildlife, livestock, and human settlements increase the likelihood of acquiring and transmitting antimicrobial-resistant microorganisms. Additionally, factors such as roosting behavior, dietary diversity, and unique physiological traits may contribute to their efficiency as reservoirs and disseminators of these pathogens [30]. Understanding the role of bats in the ecology of antimicrobial resistance is essential for assessing the risks of pathogen spillover and designing effective surveillance strategies.

### 3.1. Bacterial Isolates

**Figure 1:**
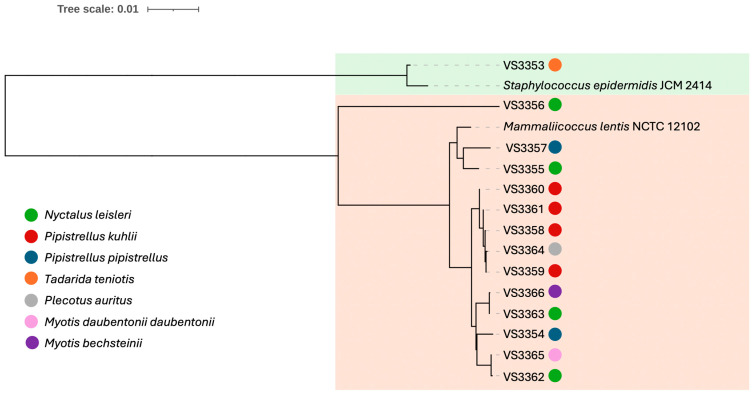
Phylogenetic analyses of *M. lentus* and *S. epidermidis* strains isolated from bats in Portugal.

Among the 105 bat samples, 13 (12.4%) *Mammaliicoccus* and only 1 *Staphyloccocus* were isolated. The isolates were identified as *M. lentus* and *S. epidermidis*. *M. lentus* isolates were recovered from six different bat species: *Nyctalus leisleri* (*n* = 4), *Pipistrellus kuhlii* (*n* = 4) *Pipistrellus pipistrellus* (*n* = 2), *Plecotus auratus*, *Myotis daubentonii daubentoniid*, and *Myotis bechsteinii*; while the one *S. epidermidis* was isolated from *Tadarida teniotis* (Supplementary Table S1). The phylogenetic tree of the *M*. *lentus* and *S. epidermidis* isolates is shown in Figure 1. The *M. lentus* strains isolated from bats of the same species appear on distinct branches of the phylogenetic tree, indicating genomic distinction.

*Staphylococcus* spp. and *Mammaliicoccus* spp. (former *Staphylococcus*) seem to be one of the most common genera of pathogenic bacteria found in bats in other studies [3,31,32]. In fact, Ferreira et al. highlighted that *Bartonella* spp., *Leptospira* spp., and *Staphylococcus* spp. were among the most frequently detected pathogens, with their occurrence showing a notable rise over the years [31]. Various studies have highlighted the presence of *Staphylococcaceae* in bats across different regions. In an Australian study examining semen, urethral, and preputial swabs from *Pteropus* bats, *Streptococcus* and *Staphylococcus* were among the most frequently detected bacterial genera [33]. In Brazil, *M. sciuri* was the most commonly isolated species, detected in 66.7% (6/9) of bat species, along with *Staphylococcus aureus*, *Staphylococus saprophyticus, Staphylococus warneri*, *Staphylococus xylosus*, *Staphylococus kloosii*, *Staphylococus epidermidis*, *Staphylococus haemolyticus*, and *Staphylococus nepalensis* [28]. Similarly, *Pipistrellus abramus* in Asia was found to harbor *S. nepalensis* [25], while *Pipistrellus nathusii* was associated with *S. warneri, S. xylosus*, *M. sciuri*, *M. lentus*, and *S. equorum* [34]. In Mexico, *S. epidermidis* was detected in the interscapular dorsal patch of bats [35].

European studies present a varied picture regarding the presence of Staphylococcus and *Mammaliicoccus* in bats. In Italy, research on species such as *Tadarida teniotis*, *Miniopterus schreibersi*, *M. capaccinii*, *M. daubentonii*, *P. kuhlii*, and *M. myotis* revealed a diverse microbiota, yet no detection of *Staphylococcus* spp. or *Mammaliicoccus* spp. was noted [36]. However, in the Netherlands, *S. capitis* was identified in *M. emarginatus* [37]. Slovakian studies detected *S. equorum* (2%) and *S. nepalensis* (96%) in *Rhinolophus hipposideros* [38], while in Serbia, *S. epidermidis* was found in fecal samples [39]. Additionally, Slovakia also reported the presence of *M. lentus* and *M. sciuri* in bat feces [40]. *Staphylococcaceae* species such as *S. xylosus*, *S. kloosii*, *S. simiae*, *S. aureus*, and *M. sciuri* have also been reported in bats in the United Kingdom [41] and Spain [42]. *S. nepalensis* is frequently detected in bats, suggesting that it may constitute part of their natural microbiota. Despite its common occurrence in previous studies, our analysis did not identify any *S. nepalensis* isolates, diverging from what has been previously reported in the literature [24,25,28,43]. On the other hand, *M. lentus* appears to be less frequently encountered in bats. However, its presence has been documented in various studies, including detections in lesser horseshoe bats in India [44], *Saccopteryx bilineata* in Costa Rica [45], and both *M. myotis* and *M. blythii* in Slovakia [40]. While its occurrence in bats remains relatively infrequent, *M. lentus* has been associated with a variety of infections, such as endocarditis, peritonitis, septic shock, urinary tract infection, sinusitis, splenic abscess, and wound infections [46]. *M. lentu’s* relevance in human medicine has been increasing due to its reported association with wound infections, emphasizing the need for further research into its potential zoonotic impact.

Our study, carried out during the summer months, aligns with prior findings indicating that bat activity peaks during this season, coinciding with increased food availability. This period is particularly significant for pregnant and lactating females as it provides optimal conditions for foraging [47,48]. Such heightened activity likely fosters greater interaction with other species and environmental elements, which may explain the higher prevalence of *Staphylococcaceae* isolates observed in our samples.

### 3.2. Resistance, Virulence, and Molecular Typing

Regarding the antimicrobial resistance of the isolates, only three isolates (*S. epidermidis*, *M. lentus* VS3355, and *M. lentus* VS3362) showed a multidrug resistance profile. *S. epidermidis* showed resistance to clindamycin, trimethoprim-sulfamethoxazole, and fusidic acid, which were conferred by the *mph*(C), *msr*(A), and *dfr*C (Table 1). *S. epidermidis* also carried other genes associated with antimicrobial resistance, such as *fos*B, *mgr*A, and *nor*C. All *M. lentus* isolates were resistant to clindamycin, which was encoded by the *sal*B gene present in all isolates. One isolate (VS3355) showed resistance to tetracycline and carried the *tet*(K) gene. The same isolate carried the *str* gene, which confers resistance to streptomycin, but resistance to this antibiotic was not tested phenotypically. Even though 8 of the 13 isolates showed resistance to fusidic acid, all lacked the known resistance genes.

The *mph* genes encode the macrolide phosphotransferase, which mediates specific resistance to macrolides [49,50]. Studies have shown that the gene products of the *mph*(C) variants in *M. lentus*, *M. sciuri*, and *S. cohnii* do not confer resistance to macrolides [50,51,52]. However, not very common, *mph*(C) has been detected in *M. lentus* strains isolated from wild animals in natural environments [53,54]. Additionally, the *mph*(C) gene, which confers resistance to macrolides, does not mediate resistance to lincosamides [55]. The exception to this pattern was the *S. epidermidis* isolate, which not only displayed phenotypic resistance to clindamycin but also to erythromycin. Unlike the other isolates, this strain harbored both the *mph*(C) and *msr*(A) genes. This is relevant because *mph*(C) is often found in association with *msr*(A), and while *mph*(C) alone confers only low-level macrolide resistance, the presence of *msr*(A) may enhance the resistance profile, explaining the erythromycin resistance observed in this isolate [56]. All *M. lentus* isolates carried the *salB* gene, which confers resistance to lincosamides and class A streptogramins. Although not frequently reported, this gene has previously been identified in *M. lentus* strain K169, isolated from cows with mastitis in India [57]. Research carried out in Spain and Slovakia reported that all *Staphylococcus* isolates from bats were resistant to erythromycin, with high levels of resistance also observed for streptomycin and tetracycline [42,58]. In our study, only one isolate showed resistance to tetracycline, mediated by the *tet*(K) gene, which encodes efflux proteins [59]. Tetracycline resistance is also frequently detected in different CoNS species [55,60]. Twelve of the fourteen isolates showed phenotypic resistance to fusidic acid despite the absence of the *fus* genes associated with this antibiotic in *Staphylococcaceae*. The phenotypic resistance observed in these isolates may be mediated by alternative mechanisms or unidentified resistance determinants. Similar results were obtained in other studies in mammaliicoccal and staphylococcal isolates from various animals, including wildlife [24,61,62,63]. In *Staphylococcaceae*, resistance to trimethoprim is associated with the presence of various *dfr* genes [64]. The *dfr*C gene was detected in *S. epidermidis* in this study; however, information on the presence of *dfr* genes in mammaliicocci is still scarce [65].

The *S. epidermidis* isolate harbored the virulence genes *hld* and *ica*C, while all *M. lentus* lacked virulence genes. The *S. epidermidis* isolate carried one biocide resistance, *qacA,* which codes for multidrug efflux pumps, providing resistance to quaternary ammonium compounds and intercalating agents [66]. In vitro, studies suggest a link between *qacA* and chlorhexidine tolerance in *S. aureus*. Nevertheless, the clinical significance of this low-level tolerance remains uncertain [67]. The Qac efflux pump genes are primarily found on plasmids, facilitating their potential spread among bacterial strains. However, *qacA* has only been identified on large, non-conjugative multidrug resistance plasmids, which lack the necessary *tra* genes for conjugative transfer [68]. Nevertheless, the detection of the *qacA* gene in staphylococci from bats raises potential public health concerns due to its plasmid-associated nature. This gene could potentially transfer to more pathogenic staphylococcal species, such as *S. aureus*, via horizontal gene transfer. Both *S. epidermidis* and *M. lentus* VS3355 isolates carried one heavy metal resistance gene each. *S. epidermidis* carried the *arsB*, which confers resistance to arsenic, while M. lentus harbored the *cad*D gene, which is employed by several bacteria for cadmium tolerance [69]. The presence of heavy metals in the environment has driven bacteria to develop resistance, similar to how antibiotic resistance arises from overuse. Heavy metals like arsenic, copper, and zinc can promote co-selection, indirectly enhancing antibiotic resistance. Even low levels of these metals have been shown to increase bacterial resistance to antibiotics, underscoring the link between heavy metal exposure and antimicrobial resistance [70].

Regarding molecular typing, the *S. epidermidis* strain belonged to ST297. This clone has been reported in *S. epidermidis* from healthy humans and invasive *S. epidermidis* from human infections [71,72,73,74].

### 3.3. Plasmids and Mobile Genetic Elements

The plasmid analysis by PlasmidFinder revealed two different plasmid replicon types. Plasmids were present in the *S. epidermidis* isolate (*rep*_US76_) and in one *M. lentus* (*rep*_7a_). The plasmid rep7a in *M. lentus* VS3355 harbored the *tet*(K) and *str* genes, consistent with previous reports [75,76,77]. These genes, located on plasmids, can be readily transferred between cells through conjugation, facilitating the spread of resistance. *S. epidermidis* also carried the insertion sequence ISSep3, belonging to the IS200/IS605 family. This mobile genetic element is frequently detected in *S. epidermidis* and is closely linked to the transfer of resistance genes; however, it has been poorly studied in the literature [78,79].

## 4. Conclusions

This study investigated the role of bats as reservoirs of antimicrobial-resistant *Mammaliicoccus lentus* and *Staphylococcus epidermidis*, highlighting their relevance to public health. The identification of these isolates revealed the presence of antibiotic-resistance genes, some associated with mobile genetic elements, suggesting their potential dissemination to other pathogenic bacteria. Additionally, the detection of heavy metal resistance genes reinforces the link between environmental factors and the co-selection of antimicrobial resistance. These findings underscore the importance of wildlife surveillance to better understand the ecology of antimicrobial resistance and prevent its spread. Future studies should explore the zoonotic potential of these bacteria and the mechanisms driving the acquisition and dissemination of resistance. The One Health approach remains crucial for monitoring and mitigating emerging threats to global public health.

## Figures and Tables

**Table 1:** Characteristics of bacterial isolates from bats, including resistance profiles, virulence factors, and genetic elements.

Isolate	Species	Antimicrobial Resistance		Virulence Factors	Molecular Typing	Plasmids and MGEs	Other Resistance Genes
		Phenotype	Genotype		ST		
VS3353	*S. epidermidis*	ERY, CD, SXT, FD	*mph*(C), *msr*(A), *nor*C*, mgr*A, *fos*B, *dfr*C	*icaC*, *hld*	297	*rep_US76_*, SS*ep3*	*arsB*, *qacA*
VS3354	*M. lentus*	CD, FD	*mph*(C), *sal*B				
VS3355	*M. lentus*	TET, FD	*str*, *mph*(C), *tet*(K), *sal*B			rep7a (pS194), rep7a (Cassette)	*cad*D
VS3356	*M. lentus*	CD	*mph*(C), *sal*B				
VS3357	*M. lentus*	CD, FD	*mph*(C), *sal*B				
VS3358	*M. lentus*	FD	*mph*(C), *sal*B				
VS3359	*M. lentus*	CD, FD	*mph*(C), *sal*B				
VS3360	*M. lentus*	CD, FD	*mph*(C), *sal*B				
VS3361	*M. lentus*	CD, FD	*mph*(C), *sal*B				
VS3362	*M. lentus*	PEN, CD, FD	mph(C), *sal*B, *cat*				
VS3363	*M. lentus*	CD, FD	*mph*(C), *sal*B				
VS3364	*M. lentus*	CD, FD	*mph*(C), *sal*B				
VS3365	*M. lentus*	CD, FD	*mph*(C), *sal*B, *cat*				
VS3366	*M. lentus*	CD	*mph*(C), *sal*B				

Abbreviations: ERY: erythromycin; CD: clindamycin; SXT: trimethoprim-sulfamethoxazole; FD: fusidic acid; TET: tetracycline; PEN: penicillin; ST: sequence type.

## Data Availability

The original contributions presented in this study are included in the article/Supplementary Materials. Further inquiries can be directed to the corresponding authors.

## Supplementary Materials

The following supporting information can be downloaded at: https://www.mdpi.com/article/10.3390/vetsci12040322/s1, Table S1: Bat species, capture details, and bacterial isolates identified during the study.
