# Supplementary material for: Bats as Hosts of Antimicrobial-Resistant Mammaliicoccus lentus and Staphylococcus epidermidis with Zoonotic Relevance
Source: Vet Sci. 2025 Apr 1;12(4):322. doi: 10.3390/vetsci12040322 (PMC12031343; doi:10.3390/vetsci12040322)
Supplement: Supplementary file 1 [file vetsci-12-00322-s001.zip › vetsci-3490454-supplementary.pdf]

Table S1. Bat species, capture details, and bacterial isolates identified during the study.

Bat	Capture date	Species	Sex	Age	Location	Bacterial isolate
M1	30/05/2022	*Myotis escalerai*	F	A	Vila Real	VS3354
M2	04/07/2022	*Nyctalus leisleri*	M	J	Teixedo, Azibo	
M3	04/07/2022	*Nyctalus leisleri*	M	A	Teixedo, Azibo	
M4	04/07/2022	*Nyctalus leisleri*	M	A	Teixedo, Azibo	
M5	04/07/2022	*Pipistrellus pipistrellus*	F	A	Teixedo, Azibo	VS3356
M6	04/07/2022	*Nyctalus leisleri*	M	A	Teixedo, Azibo	VS3357
M7	04/07/2022	*Nyctalus leisleri*	F	A	Teixedo, Azibo	
M8	04/07/2022	*Nyctalus leisleri*	M	A	Teixedo, Azibo	
M9	04/07/2022	*Nyctalus leisleri*	M	A	Teixedo, Azibo	
M10	04/07/2022	*Nyctalus leisleri*	F	J	Teixedo, Azibo	
M11	04/07/2022	*Nyctalus leisleri*	F	A	Teixedo, Azibo	
M12	04/07/2022	*Barbastella barbastellus*	F	A	Teixedo, Azibo	
M13	04/07/2022	*Myotis escalerai*	M	A	Teixedo, Azibo	
M14	04/07/2022	*Pipistrellus pipistrellus*	F	J	Teixedo, Azibo	
M15	04/07/2022	*Rhinolophus ferrumequinum*	F	A	Teixedo, Azibo	VS3362
M16	04/07/2022	*Pipistrellus pipistrellus*	F	A	Teixedo, Azibo	
M17	04/07/2022	*Plecotus auritus*	M	A	Teixedo, Azibo	
M18	04/07/2022	*Pipistrellus pipistrellus*	F	A	Teixedo, Azibo	
M19	04/07/2022	*Pipistrellus pipistrellus*	F	A	Teixedo, Azibo	
M20	04/07/2022	*Miniopterus schreibersii*	F	A	Teixedo, Azibo	
M21	18/07/2022	*Pipistrellus kuhlii*	F	A	Carlão, Alijó	
M22	18/07/2022	*Pipistrellus kuhlii*	M	J	Carlão, Alijó	
M23	18/07/2022	*Pipistrellus kuhlii*	M	A	Carlão, Alijó	
M24	18/07/2022	*Pipistrellus kuhlii*	F	J	Carlão, Alijó	
M25	18/07/2022	*Pipistrellus kuhlii*	F	A	Carlão, Alijó	
M26	18/07/2022	*Pipistrellus kuhlii*	M	J	Carlão, Alijó	
M27	18/07/2022	*Pipistrellus kuhlii*	F	A	Carlão, Alijó	
M28	18/07/2022	*Pipistrellus kuhlii*	F	A	Carlão, Alijó	
M29	18/07/2022	*Pipistrellus kuhlii*	M	J	Carlão, Alijó	
M30	18/07/2022	*Pipistrellus kuhlii*	F	J	Carlão, Alijó	VS3361
M31	18/07/2022	*Tadarida teniotis*	F	A	Carlão, Alijó	
M32	18/07/2022	*Tadarida teniotis*	F	A	Carlão, Alijó	
M33	18/07/2022	*Tadarida teniotis*	M	A	Carlão, Alijó	
M34	18/07/2022	*Tadarida teniotis*	F	A	Carlão, Alijó	
M35	18/07/2022	*Tadarida teniotis*	M	J	Carlão, Alijó	

M36	18/07/2022	*Tadarida teniotis*	F	A	Carlão, Alijó	
M37	18/07/2022	*Tadarida teniotis*	F	A	Carlão, Alijó	
M38	18/07/2022	*Tadarida teniotis*	F	A	Carlão, Alijó	
M39	25/07/2022	*Pipistrellus pipistrellus*	F	A	Rio de Onor, Bragança	
M40	25/07/2022	*Pipistrellus pipistrellus*	F	P	Rio de Onor, Bragança	
M41	25/07/2022	*Pipistrellus pipistrellus*	M	P	Rio de Onor, Bragança	
M42	25/07/2022	*Plecotus austriacus*	M	A	Rio de Onor, Bragança	VS3358
M43	25/07/2022	*Hypsugo savii*	M	A	Rio de Onor, Bragança	
M44	25/07/2022	*Myotis daubentonii*	M	A	Rio de Onor, Bragança	
M45	25/07/2022	*Plecotus austriacus*	M	A	Rio de Onor, Bragança	
M46	26/07/2022	*Pipistrellus pipistrellus*	M	A	Lamas de Olo, Alvão	
M47	26/07/2022	*Myotis mystacinus*	F	P	Lamas de Olo, Alvão	
M48	26/07/2022	*Myotis mystacinus*	F	J	Lamas de Olo, Alvão	
M49	26/07/2022	*Myotis myotis*	M	A	Lamas de Olo, Alvão	VS3356
M50	26/07/2022	*Plecotus austriacus*	F	A	Lamas de Olo, Alvão	
M51	11/08/2022	*Rhinolophus mehelyi*	M	A	Torre, Évora	
M52	11/08/2022	*Rhinolophus mehelyi*	M	A	Torre, Évora	
M53	11/08/2022	*Rhinolophus mehelyi*	M	A	Torre, Évora	
M54	11/08/2022	*Miniopterus schreibersii*	-	-	Torre, Évora	
M55	11/08/2022	*Miniopterus schreibersii*	-	-	Torre, Évora	VS3359
M56	11/08/2022	*Miniopterus schreibersii*	-	-	Torre, Évora	VS3363
M57	11/08/2022	*Miniopterus schreibersii*	-	-	Torre, Évora	
M58	11/08/2022	*Myotis myotis*	-	-	Torre, Évora	
M59	11/08/2022	*Rhinolophus mehelyi*	M	A	Torre, Évora	
M60	11/08/2022	*Rhinolophus mehelyi*	F	A	Torre, Évora	
M61	15/09/2022	*Pipistrellus pipistrellus*	M	A	Aldeia Velha, Sabugal	
M62	15/09/2022	*Plecotus austriacus*	F	A	Aldeia Velha, Sabugal	

M63	15/09/2022	*Plecotus austriacus*	M	J	Aldeia Velha, Sabugal	
M64	15/09/2022	*Plecotus austriacus*	M	A	Aldeia Velha, Sabugal	
M65	15/09/2022	*Plecotus austriacus*	F	A	Aldeia Velha, Sabugal	
M66	15/09/2022	*Plecotus austriacus*	F	A	Aldeia Velha, Sabugal	
M67	15/09/2022	*Plecotus austriacus*	F	A	Aldeia Velha, Sabugal	
M68	15/09/2022	*Plecotus austriacus*	F	A	Aldeia Velha, Sabugal	
M69	15/09/2022	*Plecotus austriacus*	F	A	Aldeia Velha, Sabugal	
M70	15/09/2022	*Plecotus austriacus*	F	A	Aldeia Velha, Sabugal	
M71	15/09/2022	*Plecotus austriacus*	F	A	Aldeia Velha, Sabugal	
M72	15/09/2022	*Plecotus austriacus*	F	A	Aldeia Velha, Sabugal	
M73	15/09/2022	*Plecotus austriacus*	M	A	Aldeia Velha, Sabugal	
M74	15/09/2022	*Plecotus austriacus*	F	J	Aldeia Velha, Sabugal	
M75	15/09/2022	*Plecotus austriacus*	F	A	Aldeia Velha, Sabugal	
M76	15/09/2022	*Plecotus austriacus*	M	A	Aldeia Velha, Sabugal	
M77	15/09/2022	*Plecotus auritus*	M	A	Aldeia Velha, Sabugal	VS3364
M78	15/09/2022	*Plecotus austriacus*	F	A	Aldeia Velha, Sabugal	
M79	15/09/2022	*Plecotus austriacus*	F	A	Aldeia Velha, Sabugal	
M80	15/09/2022	*Pipistrellus pipistrellus*	F	A	Aldeia Velha, Sabugal	
M81	31/08/2022	*Pipistrellus pipistrellus*	M	A	Campeã, Vila Real	
M82	31/08/2022	*Pipistrellus pipistrellus*	F	A	Campeã, Vila Real	
M83	31/08/2022	*Hypsugo savii*	M	A	Campeã, Vila Real	
M84	31/08/2022	*Nyctalus leisleri*	M	A	Campeã, Vila Real	
M85	31/08/2022	*Pipistrellus pipistrellus*	F	A	Campeã, Vila Real	
M86	20/09/2022	*Myotis escalerai*	M	A	Minas de St. Adrião, Miranda Douro	VS3360

M87	20/09/2022	*Myotis escalerai*	M	A	Minas de St. Adrião, Miranda Douro	
M88	20/09/2022	*Myotis escalerai*	M	A	Minas de St. Adrião, Miranda Douro	
M89	20/09/2022	*Myotis escalerai*	M	A	Minas de St. Adrião, Miranda Douro	
M90	20/09/2022	*Myotis escalerai*	M	A	Minas de St. Adrião, Miranda Douro	
M91	20/09/2022	*Rhinolophus ferrumequinum*	M	A	Minas de St. Adrião, Miranda Douro	
M92	20/09/2022	*Myotis bechsteinii*	M	A	Minas de St. Adrião, Miranda Douro	
M93	20/09/2022	*Myotis bechsteinii*	M	A	Minas de St. Adrião, Miranda Douro	
M94	20/09/2022	*Pipistrellus pipistrellus*	F	A	Minas de St. Adrião, Miranda Douro	
M95	20/09/2022	*Myotis daubentonii daubentonii*	M	A	Minas de St. Adrião, Miranda Douro	VS3365
M96	20/09/2022	*Myotis bechsteinii*	M	A	Minas de St. Adrião, Miranda Douro	
M97	20/09/2022	*Myotis bechsteinii*	M	A	Minas de St. Adrião, Miranda Douro	
M98	20/09/2022	*Pipistrellus pipistrellus*	M	A	Minas de St. Adrião, Miranda Douro	
M99	20/09/2022	*Myotis bechsteinii*	M	A	Minas de St. Adrião, Miranda Douro	
M100	20/09/2022	*Myotis escalerai*	M	A	Minas de St. Adrião, Miranda Douro	VS3353
M101	20/09/2022	*Myotis daubentonii nat*	M	A	Minas de St. Adrião, Miranda Douro	
M102	20/09/2022	*Myotis daubentonii nat*	M	A	Minas de St. Adrião, Miranda Douro	
M103	20/09/2022	*Myotis daubentonii daubentonii*	M	A	Minas de St. Adrião, Miranda Douro	

M104	20/09/2022	*Rhinolophus euryale*	M	A	Minas de St. Adrião, Miranda Douro	
M105	20/09/2022	*Myotis bechsteinii*	M	A	Minas de St. Adrião, Miranda Douro	VS3366

Abreviations: M: male; F: female; A: adult; J: juvenile; P: pup.
